# Hospitalizations in patients with systemic lupus erythematosus: updated analyses from 2006 to 2011

**DOI:** 10.1186/ar3993

**Published:** 2012-09-27

**Authors:** K Chan, A Dekis, AE Clarke, CA Pineau, E Vinet, E Nashi, S Bernatsky

**Affiliations:** 1 McGill University, Montreal, QC, Canada; 2 McGill University Health Centre, Montreal, QC, Canada

## Background

Health resource use is believed to be significant in patients with systemic lupus erythematosus (SLE), but there is a lack of data especially in Canadian patients regarding the reasons why persons with SLE require hospitalization and the rates of hospitalization compared with the general population. Our objective was to provide recent estimates for hospitalization rates and reasons for admission, in a clinical SLE cohort.

## Methods

We evaluated data from patients with SLE followed at the McGill University Health Center Lupus Clinic. Information on disease activity, drug exposure, health outcomes, and hospitalizations by self-report were collected from annual research visits. The hospitalization rates of the SLE patients were generated. We compared this with the Canadian general population by calculating the standardized incidence ratio (SIR), which represents the ratio of the number of events observed in the SLE cohort to the number of events that would be expected based on the age-specific and sex-specific Canadian general population hospitalization rates.

## Results

Over the interval studied, 350 patients (325 female, 25 male) provided 1,261 person-years of follow-up. There were 163 reported admissions with an incidence of 12.8 hospitalizations per 100 person-years (12.4 in females, 19.5 in males). SLE-related causes (for example, flares) accounted for the highest proportion of hospitalization (22.7%), followed by infections (20.2%), surgery (14.7%), childbirth (11.7%) and cardiovascular reasons (11.0%). The overall SIR was 1.73 (95% CI = 1.48 to 2.02). Stratified by sex, the SIR was 2.87 (95% CI = 1.67 to 4.60) for males and 1.39 (95% CI = 1.18 to 1.64) for females. However, stratifying further by age, female SLE patients aged >65 actually underwent fewer hospitalizations than expected, based on age/sex-specific general population rates (SIR = 0.35; 95% CI = 0.17 to 0.64). In male SLE patients over 65, there were no hospitalizations (compared with 1.48 expected events), and the confidence interval (95% CI = 0.0 to 2.49) around the SIR was very imprecise in this demographic, due to the relatively low number of older males in our cohort.

## Conclusion

We documented high rates of hospitalization in our SLE patients, particularly for male patients. Hospitalizations were often due to SLE-related reasons and infections. Female SLE patients over the age of 65 were shown to have a much lower hospitalization rate compared with the general population, which may be due to a survivorship bias. Further work on the variables affecting hospitalizations in SLE patients is in progress.

